# Factors Associated With Clinical Responses to Spinal Manipulation in Patients With Non-specific Thoracic Back Pain: A Prospective Cohort Study

**DOI:** 10.3389/fpain.2021.742119

**Published:** 2022-01-06

**Authors:** Mégane Pasquier, James J. Young, Arnaud Lardon, Martin Descarreaux

**Affiliations:** ^1^Department of Anatomy, Université du Québec à Trois-Rivières, Trois-Rivières, QC, Canada; ^2^Institut Franco-Européen de Chiropraxie, Toulouse, France; ^3^Center for Muscle and Joint Health Department, University of Southern Denmark, Odense, Denmark; ^4^Department of Research, Canadian Memorial Chiropractic College, Toronto, ON, Canada; ^5^Department of Human Kinetics, Université du Québec à Trois-Rivières, Trois-Rivières, QC, Canada

**Keywords:** manual therapies, spine pain, observational study, association, comfort, prognosis

## Abstract

**Introduction:** The management of musculoskeletal disorders is complex and requires a multidisciplinary approach. Manual therapies, such as spinal manipulative therapy (SMT), are often recommended as an adjunct treatment and appear to have demonstrable effects on pain and short-term disability in several spinal conditions. However, no definitive mechanism that can explain these effects has been identified. Identifying relevant prognostic factors is therefore recommended for people with back pain.

**Objective:** The main purpose of this study was to identify short-term candidate prognostic factors for clinically significant responses in pain, disability and global perceived change (GPC) following a spinal manipulation treatment in patients with non-specific thoracic back pain.

**Methods:** Patients seeking care for thoracic spine pain were invited to participate in the study. Pain levels were recorded at baseline, post-intervention, and 1 week after a single session of SMT. Disability levels were collected at baseline and at 1-week follow-up. GPC was collected post-intervention and at 1-week follow-up. Biomechanical parameters of SMT, expectations for improvement in pain and disability, kinesiophobia, anxiety levels as well as perceived comfort of spinal manipulative therapy were assessed.

**Analysis:** Differences in baseline characteristics were compared between patients categorized as responders or non-responders based on their pain level, disability level, and GPC at each measurement time point. Binary logistic regression was calculated if the statistical significance level of group comparisons (responder vs. non-responders) was equal to, or <0.2 for candidate prognostic factors.

**Results:** 107 patients (62 females and 45 males) were recruited. Mean peak force averaged 450.8 N with a mean thrust duration of 134.9 ms. Post-intervention, comfort was associated with pain responder status (*p* < 0.05) and GPC responder status (*p* < 0.05), while expectation of disability improvement was associated with GPC responder status (*p* < 0.05). At follow-up, comfort and expectation of pain improvement were associated with responder GPC status (*p* < 0.05). No association was found between responder pain, disability or GPC status and biomechanical parameters of SMT at any time point.

**Discussion:** No specific dosage of SMT was associated with short-term clinical responses to treatment. However, expectations of improvement and patient comfort during SMT were associated with a positive response to treatment.

## Introduction

Musculoskeletal disorders (MSD) represent a growing public health issue for societies, with an ~30% increase in MSD-related disability observed globally since 1990 (1). The International Classification of Diseases defines musculoskeletal disorders as any conditions affecting the musculoskeletal system components, including muscles, bones, joints and associated tissues, as well as tendons and ligaments (2). Of all MSD, spinal pain syndromes are classified among the most disabling ones, low back pain alone being the leading cause of disability in 14 of the 21 regions of the world. In fact, low back pain-related years lived with disability (YLD) has increased by 52.7% over the past decade, representing 64.9 million YLD (1).

Although there are far fewer studies investigating the nature and treatment options for thoracic spine pain than for low back and cervical pain, spinal pain seems to have similar characteristics across the cervical, thoracic and lumbar regions (3, 4). In fact, thoracic pain, like cervical and lumbar pain, has been shown to generate significant disability at work and in daily life activities (4, 5). The annual prevalence of thoracic spine pain is around 15–35% in the general adult population and the 1-year prevalence can reach up to 55% in the working population (6, 7). Women are 2.5 times more likely to suffer from thoracic spine and chest pain compared to men, and musculoskeletal comorbidities are considered risk factors for thoracic spine pain. Moreover, general work-related factors have been reported. Among them, high work load, defined as the frequency of job tasks/problems, high work intensity defined as the frequency of job tasks/problems over 5 years for specific occupational groups such as drivers or stewards and psychosocial factors, such as high mental pressure, have all been identified as potential risk factors (6).

The management of spinal disorders is complex and requires a multidisciplinary approach as recommended by recent guidelines (8, 9). As a general treatment approach for spinal pain, recommendations drawn from these guidelines include the use of a patient-centered approach, education, and manual therapies as an adjunct treatment to other evidence-based treatments such as exercise, psychological therapy, and activity advice (8, 9). A systematic review investigating the effectiveness of non-invasive interventions for musculoskeletal thoracic pain concluded that there is a lack of quality studies related to the effect of non-invasive interventions on musculoskeletal thoracic pain (10). Knecht et al. investigated the various trajectories of mid-back pain and baseline risk factors for unfavorable outcomes for patients undergoing chiropractic treatment. Their results found that pain that lasts for more than 3 months before a treatment is associated with poor outcomes (11).

Manual therapies, such as spinal manipulation therapy (SMT), are recommended and appear to have demonstrable effects on spine pain intensity and short-term disability (9, 12, 13). SMT is one of the most widely used tools used by manual therapists such as chiropractors to manage spinal pain and extremity disorders (14). Spinal pain is the most common reason to see a chiropractor. While SMT is commonly used as part of a multidisciplinary approach to treat spinal pain and disability, the underlying physiological mechanisms by which it operates remain elusive.

From a biomechanical standpoint, SMT is defined as a thrust of high velocity and low amplitude delivered to the spine using a specific contact (15). It can be characterized by its force-time profile defining specific biomechanical parameters such as thrust force, thrust duration, rate of force, and preload force. However, only a few studies have investigated the association between treatment dosages defined by these parameters and frequency with clinical outcomes. Lima et al. investigated current evidence regarding the physiological responses related to SMT procedures in animal models. Results showed that SMT approaches elicit several physiological changes that alter neural, lymphatic, autonomic, genetic, and molecular responses, for which a specific dosage seems to be required for changes to be observed (16). Similarly, in a scoping review, Pasquier et al. investigated the current state of scientific knowledge regarding the effects of SMT frequency and dosage on both clinical and physiological responses. The authors found no significant effect of treatment frequency regarding clinical outcomes during and following an SMT. The review also highlighted that various dosages can influence short-term physiological responses to an SMT, but that the association between physiological responses and clinical outcomes remains to be investigated (17). Moreover, both studies reported great variability in the delivery of SMT parameters, and highlighted the need for further investigation of the SMT dose-response relationship. In 2019, Pagé et al. investigated, in a randomized control trial, the effect of SMT biomechanical parameters on the outcomes of patients with chronic thoracic spine pain. Results showed no significant dosage effects on clinical outcomes. Even if SMT-dose effects have been studied, it is still impossible to determine a specific SMT dosage or frequency that optimizes spinal manipulative treatment (18).

Dosage-focused studies usually investigate the effect of a treatment on a specific condition, but we are unaware of any studies assessing the association between dosages and responders to a single SMT treatment where responders to a treatment are determined by a minimal improvement change (MIC) in pain intensity and disability. MIC is the patient's perception of the smallest change on a patient-reported outcome measure considered to be a clinically important improvement (19).

The primary aim of the study was to explore and identify short-term candidate prognostic factors for clinically significant responses in pain, disability and global perceived change (GPC) following a spinal manipulation treatment in patients with non-specific thoracic back pain. The study was designed to identify SMT dosages-related factors associated with positive treatment responses and identify “patient profiles” of responders to spinal manipulation treatments.

## Methods

### Study Design

This is a 7-day single group prospective cohort study including 107 patients. As described in the PROGRESS series and in a framework proposed for prognostic research (20), this was an exploratory prognostic study designed to investigate variables that can be tested for association with targeted outcomes to provide a background for confirmatory studies. This article follows the recommendations of the STROBE standard to report on study results (21). This observational study was registered at clinicaltrials.gov (NCT04388007) and was approved by the ethics committee of the Université du Québec à Trois-Rivières (CER-20-265-10.02), as well as by the French Committees of Protection of Persons (19.04.27.61617-2019_45). Written and oral informed consent were obtained for all patients. Patients were recruited at chiropractic clinics of the Institut Franco-Européen de Chiropraxie (IFEC) from February 2020 to June 2021.

### Patients

To be included, patients had to fulfill the following criteria: age over 18 years old; non-specific thoracic spine pain intensity (chronic ≥ 3 months or recurrent complaint with an NRS pain score ≥3) and speak French or English. Eligibility criteria were assessed verbally in order to establish patients' eligibility to the study (22, 23). Exclusion criteria were the presence of serious thoracic spine pathology, not being eligible to spinal manipulation (if any sign of osteoporosis, vertebral fracture history, thoracic disk herniation, symptoms due to non-MSDs, pregnancy tumors, infection, neurological diseases, fractures, etc.), and radicular pain/radiculopathy.

### Baseline Evaluation

**Figure 1:**
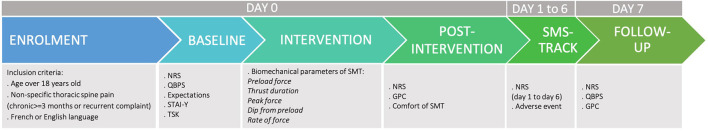
Timeline of variables' measurement. NRS = Numeric Rating Scale; QBPS = Quebec Back Pain Scale; STAI-Y = State-Trait Inventory Questionnaire; TSK = Tampa Scale of Kinesophobia; GPC = Global Perceived Change.

Patients seeking care for thoracic spine pain and meeting eligibility criteria were invited to participate in the study. They received questionnaires in consecutively numbered sealed opaque envelopes. At baseline, the following variables were collected: thoracic spine pain intensity, thoracic spine-related functional disability, kinesiophobia, anxiety, as well as expectations for improvement (details are presented in the candidate prognostic factors and clinical outcomes section). Details of the measurement timeline for the variables are presented in Figure 1. To minimize missing data, every patient who had not fully completed the questionnaires was contacted by phone or email.

### Intervention

During each treatment session, patients received a single SMT treatment for their thoracic spine pain, delivered by a final-year student in the chiropractic program. Thoracic SMT, using high velocity and low amplitude procedures, was applied between T1 and T12 owing to the patient's symptoms. The spinal segment to be manipulated was determined following a complete clinical examination and palpation of the painful area, and as agreed upon between the attending student and their clinical instructor. All SMT were performed using a posterior to anterior force application vector. SMT biomechanical parameters were recorded during the intervention using a Leander 900 Z Series treatment table (Leader Health Technologies Corporation, Port Orchard, USA) with an embedded AMTI force plate (AMTI, Watertown, MA). This device can estimate the loads transmitted during the high-velocity, low-amplitude (HVLA) manipulation and has shown reliability as well as validity in measuring force parameters (24). All transmitted forces can be computed in a xyz coordinate system using a custom-made software (MATLAB, Math-Works, Natick, USA).

### Patient Characteristics

Patients' characteristics such as sex, height, weight, age, and level of education were assessed at baseline.

### Candidate Prognostic Factors

SMT dosages were assessed using the following biomechanical parameters of spinal manipulation: peak force (N), preload force (N), force (N), thrust duration (ms), as well as rate of force application (N/s). Expectations for improvement in pain and disability, respectively, were assessed at baseline and reported via a modified version of the Patient Global Impression of Change scale. Patients answered the following questions: “On a scale of −5 to 5, how would you rate your expectations of improvement in pain/disability?”. For each construct, expectations were rated on an 11-point numeric rating scale, with 0 representing no change of pain or disability, −5 representing a deterioration of pain or disability, and +5 representing an improvement of pain or disability (25–27). Kinesiophobia was evaluated using the Tampa Scale of Kinesiophobia (TSK) at baseline. The TSK is a 17-item questionnaire that is widely used for musculoskeletal conditions. A score of 17 is the lowest possible score, and indicates no or negligible kinesiophobia, while a score of 68 is the highest possible score, indicating extreme fear of pain with movement. A score over 40 on TSK has been suggested to represent a high degree of kinesiophobia (28, 29). Anxiety was assessed pre-intervention using the State-Trait Anxiety Inventory (STAI). The questionnaire is divided into two 20-item subscales (YA and YB). The STAI-YA or State questionnaire evaluates the current state of anxiety at the time the patient fills out the questionnaire, as well as daily state of anxiety. The STAI-YB or Trait questionnaire evaluates general states of calmness, confidence, and security. A total score of the YA and YB questionnaires gives a rate of anxiety that can be classified as very low (0–35), low (36–45) moderate (46–55), high (56–65), and severe (>65) for each questionnaire (30, 31). Finally, the level of comfort during SMT was assessed following the intervention, using a 100-mm scale, a higher score indicating a very comfortable procedure and a lower score a very uncomfortable one. This criterion was identified by O'Donnell et al. as an important patient characteristic regarding SMT performance (32).

### Clinical Outcome Measures

The measurement time points for each clinical outcome measure are presented in Figure 1. Non-specific thoracic spine pain intensity was assessed using a 0–10 point Numerical Rating Scale (NRS) (33). It was also assessed every day for 7 days following the intervention using a web-based platform (SMS-FACTOR, Infomotiv SASU, France) (34). Patients answered the following question: “Did you experience any thoracic pain today?” If they answered positively, a second question was sent: “On a 0 to 10 scale, 0 being no pain and 10 being severe pain, how much would you rate this pain today?” and a call was made for any pain above pain at baseline to list any adverse event (such as muscle soreness, increase of pain, stiffness). Non-specific thoracic spine pain intensity was assessed at baseline, immediately following SMT, and at the 1-week follow-up. Because there are no MIC estimates available for thoracic spine pain, it was decided to use low back pain MIC estimates. Responders to SMT were established using a cutoff of ≥30% reduction from baseline pain scores (35).

Disability was assessed using the Quebec Back Pain Disability Scale (QBPS) (36). This questionnaire evaluates how back pain affects patients' daily life. The minimum score is 0 and the maximum score is 100, with higher scores representing greater disability. Disability was assessed at baseline and at the 1-week follow-up. As with thoracic spine pain, no disability MIC estimates are available for thoracic spine pain populations, therefore low back pain MIC estimates were used. Responders to SMT were considered using a cutoff of ≥30% reduction from the baseline of disability scores (35).

The global perceived change (GPC) was assessed post-intervention immediately following SMT and at 1-week follow-up using the following question: “How is your thoracic pain now, compared to before you entered this study?” using an 11-point score scale. A higher score meant that the pain had improved, and a lower score meant that the pain had worsened. Kamper et al. states that for an 11-point scale for GPC, any change >1.35 points is considered clinically important, and every change of 2 points or more is considered a clinically meaningful change (26).

### Statistical Analyses

#### Descriptive Analysis

The patients' baseline characteristics, spinal manipulation biomechanical parameters and clinical outcomes were calculated and presented as means and SD for continuous variables when normally distributed. Median and interquartile range were used for non-normally distributed data whereas proportions were used for categorical variables. The number and proportion of patients reporting clinically significant improvement in, respectively, (1) pain intensity (post-intervention and follow-up), (2) GPC (post-intervention and follow-up), and (3) disability level (follow-up) were calculated. Comparisons between patients who completed the study at follow-up and those who did not were performed using *T*-test or Wilcoxon rank-sum test for continuous variables (according to data distribution).

#### Responders and Non-responders' Analyses

Differences in baseline characteristics between patients categorized as responders or non-responders, based on their pain intensity level, disability level, and GPC, were compared at each time point using a *t*-test or the Wilcoxon rank-sum test for continuous variables (according to data distribution), whereas the chi-square test was used for categorical variables. To assess the strength of association with responders' status, candidate prognostic factors were included in a binary logistic regression if the group comparisons (responders vs. non-responders) level of statistical significance was equal to, or <0.2. Strength of associations were reported as odds ratios (OR), 95% confidence intervals, *p*-value and *r*^2^. An alpha level of 0.05 was used to determine statistical significance. This is an exploratory study where every variable was considered as a candidate prognostic factor and accordingly, no confounder was pre-established (37, 38). In the approach chosen, the first step is to identify significant association between variables that could be tested in a multiple confirmatory phase (38).

## Results

### Sample Characteristics

**Figure 2:**
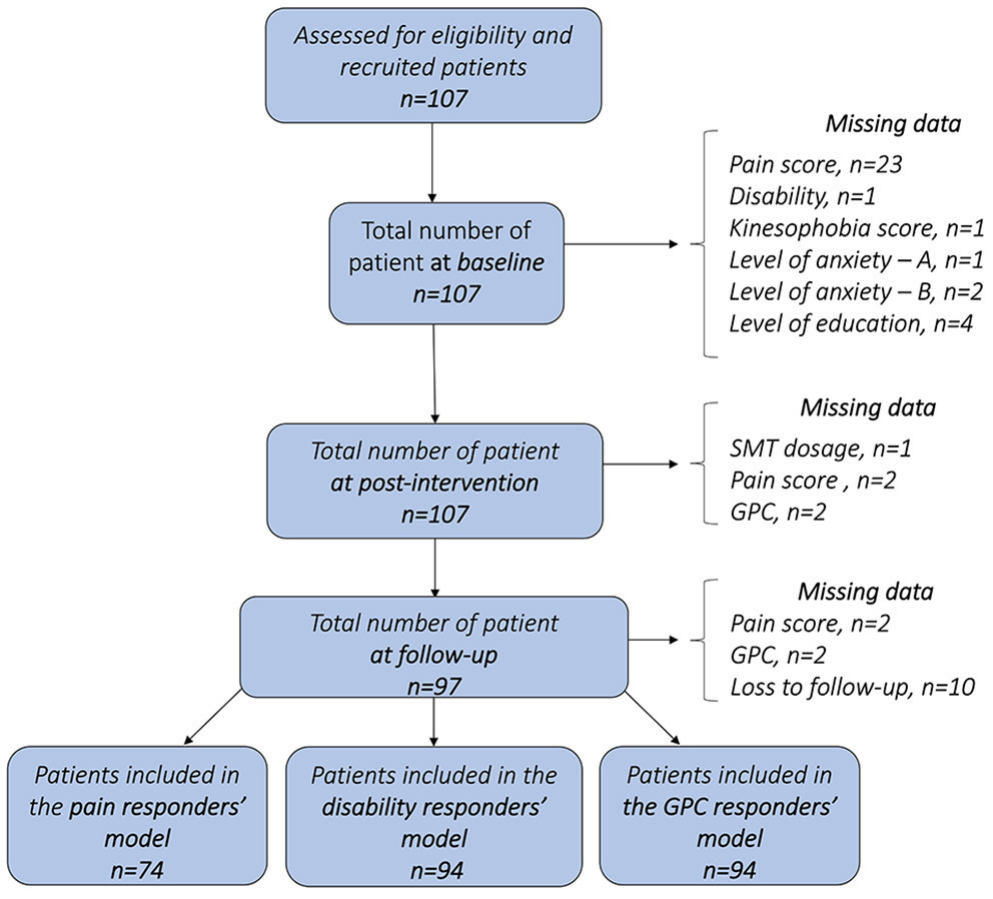
Flow diagram of included patients and data, at each measurement time point. One given patient may have missing data for one outcome and still be included in the next stages of the study. (GPC = Global Perceived Change; SMT = Spinal Manipulative Therapy).

One hundred and seven patients (62 females and 45 males) with a mean age of 32.3 years were included after meeting the pre-assessment criteria that included scoring 3 or more on the NRS pain scale (verbal). As twenty-three patients did not properly complete the NRS pain score at baseline assessment, their baseline pain intensity values were therefore excluded from the pain responders' model. At baseline, mean pain intensity and disability level averaged 4.89 (±1.7) and 15.6 (±13.6), respectively. Among all patients, 97 completed the follow-up assessments (response rate = 89%). Tests results for independent variables showed no significant differences between patients who completed the follow-up questionnaires and those who did not for baseline characteristics (Table 1). Height, weight, kinesiophobia, pain intensity at post-intervention, comfort perceived by the patients, total peak force, as well as the rate of force were normally distributed. A flow diagram of included patients and data, at each measurement time point is presented in Figure 2.

**Table 1:** Baseline characteristics of the entire sample.

	**Entire sample**	**Study completed**	**Study incompleted**	***p***
	**(*n =* 107)**	**(*n =* 95)**	**(*n =* 12)**	
**Age** (y), median (IQR)	28 (13)	27 (10)	34 (27)	0.12^+^
**Height** (cm), mean (± SD)	169.9 (±9.8)	169.9 (±9.8)	169.7 (±10.1)	0.93^−^
**Weight** (kg), mean (± SD)	68.8 (±14.2)	68.5 (±14.2)	71.2 (±14.8)	0.92^−^
**Female/Male** *(%)*	57.9/42.1	57.9/42.1	58.3/41.7	0.98^x^
**Body mass index** (kg/m^2^), median (IQR)	23.4 (4.3)	23.4 (4.2)	23.6 (6.5)	0.32^+^
**Level of education** (*n %*)				
Professional degree Secondary school Upper secondary school University degree Missing	6.8% 2.9% 21.4% 68.9% 3.7%	7.4% 3.2% 18.9% 69.5% 1.1%	0.0% 0.0% 33.3% 41.7% 25.0%	0.30^◦^
**Expectation of improvement in pain** median (IQR), (−5 to 5)	4 (2)	4 (2)	4 (2)	0.89^+^
**Expectation of improvement in disability** median (IQR) (−5 to 5)	3.5 (1.9)	4 (1.9)	3 (1.5)	0.21^+^
**Kinesiophobia–Tampa Scale** mean (± SD), (/68)	29.5 (±11.1)	29.2 (±11.1)	31.6 (±11.3)	0.49^−^
**Level of anxiety –STAI YA** *median* (IQR), (/100) Very low (*n*%) Low (*n*%) Moderate (*n*%) High (*n*%) Severe (*n*%) Missing (*n*%)	34 (13) 51% 30% 11% 5% 2% 1%	34 (15) 51% 31% 12% 5% 2% 0%	33 (9) 58% 25% 8% 0% 0% 8%	0.66^**+**^
**Level of anxiety –STAI YB** median (IQR), (/100) Very low (*n*%) Low (*n*%) Moderate (*n*%) High (*n*%) Severe (*n*%) Missing (*n*%)	38 (14) 37% 38% 25% 5% 3% 2%	39 (14) 35% 31% 25% 5% 3% 1%	35 (15) 58% 8% 25% 0% 0% 8%	0.30^**+**^
**Pain—NRS** median (IQR), (/10) Missing (*n*)	5 (2.6) 23	4.76 (2.7) 12	5 (2.5) X	0.69^**+**^
**Disability—QBPS** median (IQR), (/100) Missing (*n*)	12 (15) 1	12 (15) X	8.5 (18) X	0.36^**+**^

^*^If significant values;

+ *Wilcoxon rank-sum test*;

− *Ttest*;

X *chi2*;

◦ *Fischer*.

*n = number of patients; SD = Standard Deviation; STAI = State-Trait-Anxiety Inventory; NRS = Numeric Rating Scale; QBPS, Quebec Back Pain Scale; IQR = Interquartile Range*.

*Mean (± SD) are presented for parametric test and Median (IQR) are presented for non-parametric test*.

### Biomechanical Characteristics of SMT

Out of 107 thoracic SMT provided by chiropractic students in their final year of clinical training, 106 were recorded. Data from one SMT was not available due to technical difficulties during data collection. Mean peak force averaged 450.8 N (SD ± 156.3), with a mean time-to-peak of 134.9 ms (±41.9). SMT force-time profile characteristics are presented in Table 2.

**Table 2:** Biomechanical parameters of spinal manipulation (*n* = 106).

**Biomechanical parameters**	**Mean (±SD)**
Preload force (*N*)	185.6 (±188.3)
Peak force (*N*)	450.6 (±155.5)
Thrust duration (ms)	134.9 (±41.7)
Drop in preload (*N*)	34.7 (±40.3)
Rate of force (*N*/*s*)	2,364.1 (±864.9)

*SD = Standard Deviation*.

### Description of Outcome Variables

Mean pain intensity at baseline, at post-intervention, and at follow-up averaged respectively 4.9 (±1.7), 3.2 (±2.3), and 2.6 (±2.2). A decrease of −1.1 (±2.05) and −1.9 (±2.3) of the mean pain intensity change was observed at post-intervention and at follow-up. Means of each outcome measured at baseline, post-intervention and follow-up are presented in Table 3.

**Table 3:** Description of outcome measures.

	**Baseline**	**Post-intervention**	**Mean change at post-intervention**	**Follow-up**	**Mean change at follow-up**
**Pain—NRS (/10)**					
Sample (*n*)	84	105	82	95	74
Mean (±SD)	4.9 (±1.7)	3.2 (±2.3)	−1.1 (±2.05) [95% CI −1.5 to 0.6]	2.6 (±2.2)	−1.9 (±2.3) [95% CI −2.5 to 1.4]
Missing values (*n*)	23	2	25	12	33
**Disability-QBPS (/100)**					
Sample (n)	106			95	94
Mean (±SD)	15.6 (±13.6)	X	X	11.7 (±12.2)	−3.7 (±8.3) [95% CI −5.4 to 2.1]
Missing values (*n*)	1			12	13
**GPC (−5** **+5)**					
Sample (*n*)		105		95	
Mean (±SD)	X	2.3 (±1.6)	X	2.4 (±1.7)	X
Missing values (*n*)		2		12	

*n = number of patients; SD = Standard Deviation; STAI = State-Trait-Anxiety Inventory; NRS = Numeric Rating Scale; QBPS = Quebec Back Pain Scale; GPC = Global Perceived Change; CI = Confidence Interval*.

### Candidate Prognostic Factors of Pain Responder Status

Thirty-one patients (37.8%) were classified as responders based on pain improvement after the procedure, and 49 patients (66.2%) at follow-up (Table 4). Results of *T*-test or Wilcoxon rank sum tests for all independent variables and pain responder status are presented in Supplementary File 1. For pain responder status at post-intervention, results showed differences between groups for comfort of SMT (*p* < 0.001). At follow-up, results showed differences between responders for expectation of disability improvement (*p* = 0.036). The strength of these associations was assessed using univariate models of candidate prognostic factors associated with pain responder status at post intervention and follow-up are presented in Table 5. Comfort of SMT was associated with pain responder status at post-intervention (OR = 1.542; [95% CI 1.192–1.996], *p* = 0.017, *r*^2^ = 0.1282). Expectation of disability improvement was associated with pain responder status at follow-up (OR = 1.622; [95% CI 1.058–2.485], *p* = 0.026, *r*^2^ = 0.0570). Pain change at post-intervention was associated with pain responder status at follow-up (OR = 1.381; [95% CI 1.015–1.879], *p* = 0.039, *r*^2^ = 0.0545).

**Table 4:** Improvement status.

	**At post-intervention**	**At follow-up**
**Pain intensity**		
Responders *n* (%)	31 (37.8%)	49 (66.2%)
Non-responders *n* (%)	51 (62.2%)	25 (33.7%)
**Disability level**		
Responders *n* (%)	X	41 (43.6%)
Non-responders *n* (%)		53 (56.3%)
**GPC**		
Responders *n* (%)	68 (64.7%)	68 (72.3%)
Non-responders *n* (%)	37 (35.2%)	26 (27.6%)

*n = number of patients; NRS = Numeric Rating Scale; QBPS = Quebec Back Pain Scale; GPC = Global Perceived Change*.

**Table 5:** Univariate models of candidate prognostic factors associated with responders' pain status at post-intervention and follow-up (Odds Ratios, confidence intervals and *p*-values).

**Variables**	**Odds ratios [95% CI]**	***p***	***r* ^2^**
**Univariate models of candidate prognostic factors associated with responders' pain status at post-intervention**			
Comfort of SMT	1.542 [1.192–1.996]	<0.001	0.1282
**Univariate models of candidate prognostic factors associated with responders' pain status at follow-up**			
Expectation of improvement in disability	1.622 [1.058–2.485]	0.026	0.0570
Pain change at post-intervention	1.381 [1.015–1.879]	0.039	0.0545

*CI = Confidence Interval; SMT = Spinal Manipulative Therapy*.

### Candidate Prognostic Factors of Disability Responder Status

Of the 94 patients with complete data, 41 (43.6%) were classified as responders, based on improvement of their disability level at follow-up (Table 4), where no differences were found between the disability responder status and independent variables (*p* > 0.05). Baseline characteristics of the disability responder status at follow-up are presented in Supplementary File 2.

### Candidate Prognostic Factors of GPC Responder Status

Of the 105 patients with complete data, 68 (64.7%) were classified as responders, based on their improvement of global perceived change at post-intervention and out of 94, 68 patients (72.3%) at follow-up (Table 4). Results of *T*-test or Wilcoxon rank sum tests for all independent variables and GPC responder status are presented in Supplementary File 3. For GPC responder status at post-intervention, results showed differences between groups for expectation of disability improvement (*p* = 0.022) and comfort of SMT (*p* < 0.001). At follow-up, results showed differences between responders for expectation for improvement in pain (*p* = 0.005), in disability (*p* = 0.017), comfort of SMT (*p* = 0.006), as well as GPC at post-intervention (*p* < 0.001). The strength of these associations at post-intervention and follow-up are presented in Table 6. Expectation of disability improvement was associated with GPC responder status at post-intervention (OR = 1.487; [95% CI 1.074–2.058], *p* = 0.017, *r*^2^ = 0.0446), as well as at follow-up (OR = 1.726; [95% CI 1.178–2.528], *p* = 0.005, *r*^2^ = 0.0780). Comfort of SMT was associated with GPC responder status at post-intervention (OR = 1.326; [95% CI 1.106–1.588], *p* = 0.002, *r*^2^ = 0.0759) as well as at follow-up (OR = 1.305; [95% CI 1.069–1.594], *p* = 0.009, *r*^2^ = 0.0685). Expectation of pain improvement was associated with GPC responder status at follow-up (OR = 1.479; [95% CI 1.029–2.127], *p* = 0.034, *r*^2^ = 0.0453). GPC at post-intervention was also associated with GPC at follow-up (OR = 2.200; 95% CI [1.479–3.273], *p* < 0.001).

**Table 6:** Univariate models of candidate prognostic factors associated with GPC responder status at post-intervention and follow-up (Odds Ratios, confidence intervals and *p*-values).

**Variables**	**Odds ratios [95% CI]**	***p***	***r* ^2^**
**Univariate models of candidate prognostic factors associated with GPC responder status at post-intervention**			
Expectation of improvement in disability	1.487 [1.074–2.058]	0.017	0.0446
Comfort of SMT	1.326 [1.106–1.588]	0.002	0.0759
**Univariate models of candidate prognostic factors associated with GPC responder status at follow-up**			
Expectation of improvement in pain	1.479 [1.029–2.127]	0.034	0.0453
Expectation of improvement in disability	1.726 [1.178–2.528]	0.005	0.0780
Comfort of SMT	1.305 [1.069–1.594]	0.009	0.0685
GPC score at post-intervention	2.200 [1.479–3.273]	<0.001	0.2011

*CI = Confident Interval; SMT = Spinal Manipulative Therapy; GPC = Global Perceived Change*.

No association was found between pain, disability or GPC responder status and biomechanical parameters of SMT.

### Descriptive Results of Patients' Response Profiles

**Figure 3:**
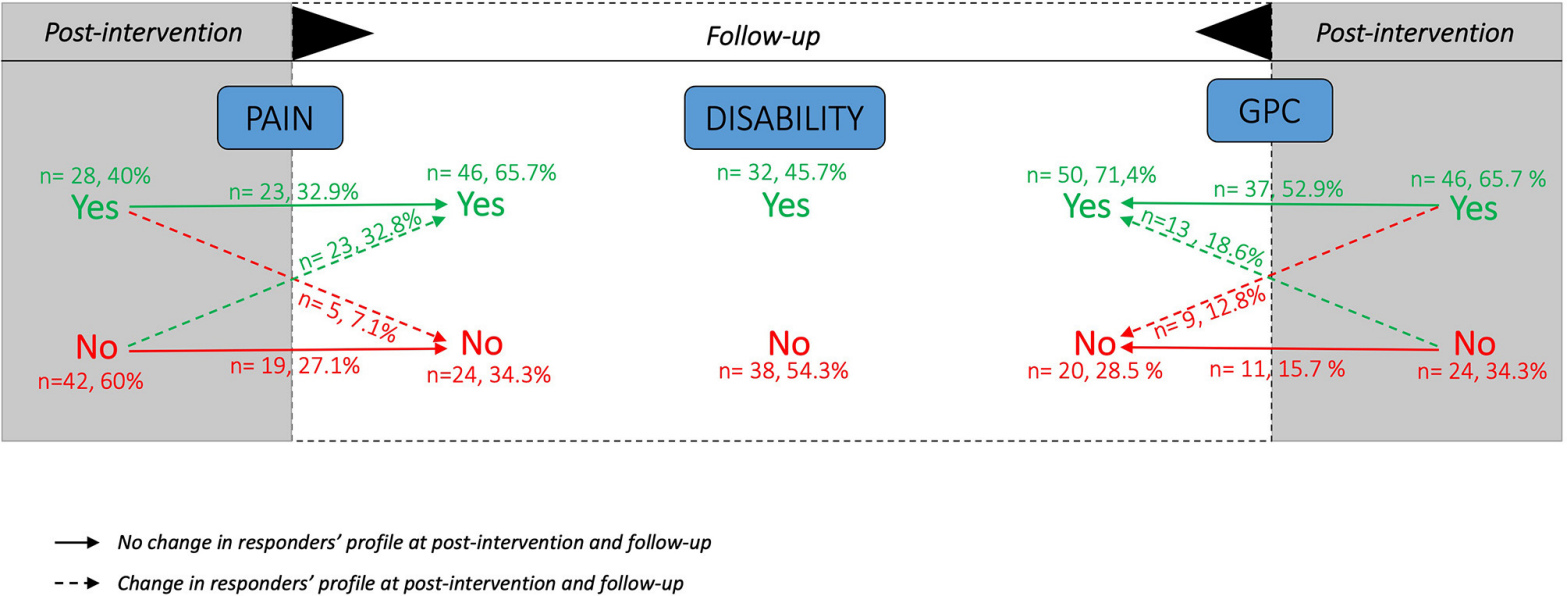
Number and proportions of responders (Yes) and non-responders (No) at each time point for each outcome measures. Based on complete data of *n* = 70 patients. GPC = Global Perceived Change; Yes = responders; No = non-responders.

**Figure 4:**
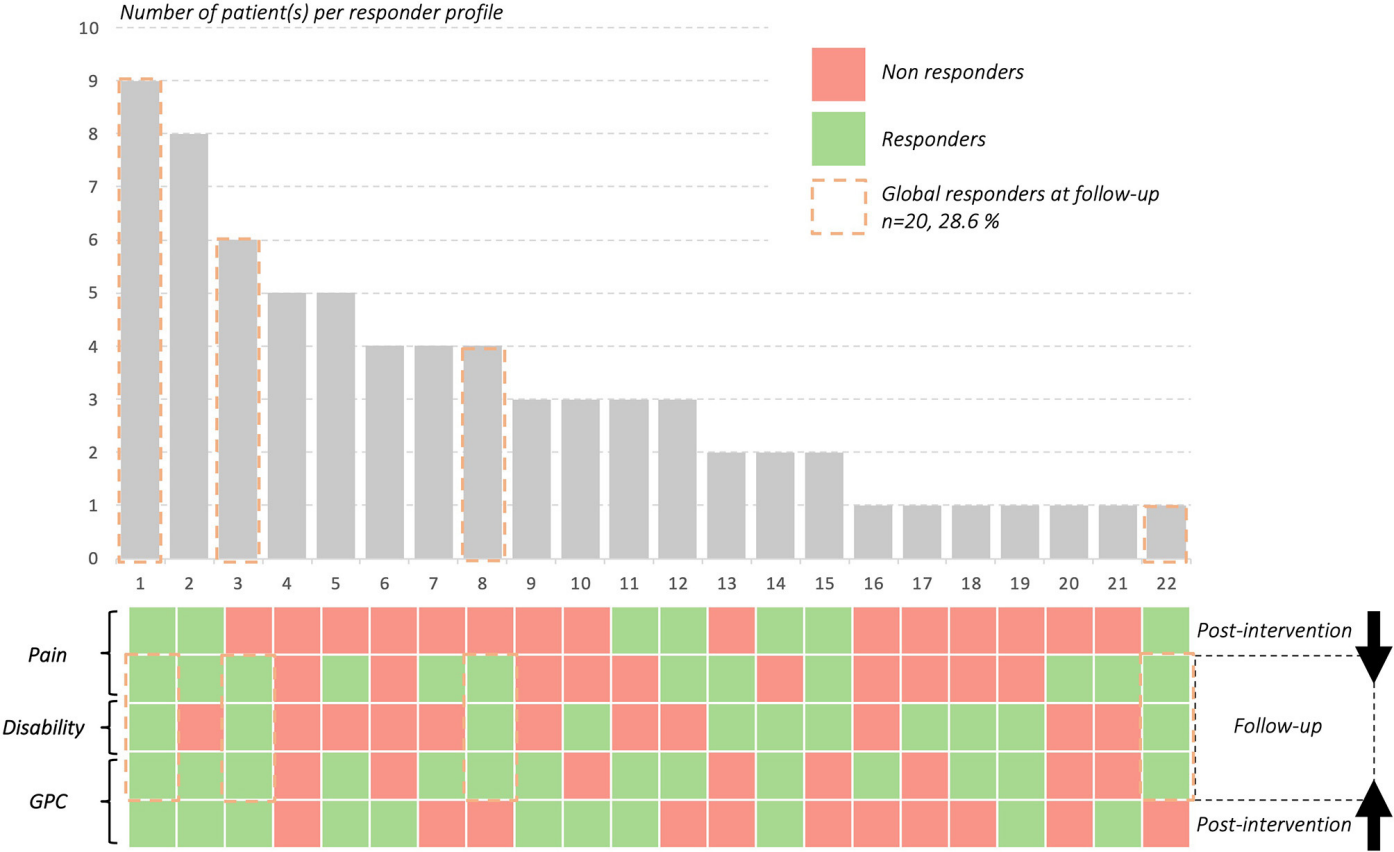
Patients' response profiles and proportions. Complete data on *n* = 70 patients with 22 patients' response profiles observed. For example, 9 patients (profile 1) were responders at all time points for each outcome whereas 5 patients were non-responders at all time points for each outcome (profile 4). GPC = GLobal Perceived Change.

Out of the 107 patients, seventy (65.4%) presented complete data. For pain responder status, 28 patients responded to treatment at post-intervention with a total of 23 still responders at follow-up. For disability responder status, 32 patients were responders to treatment at follow-up. For GPC responder status, 46 patients were responders at post-intervention with 37 still responders at follow-up. Details of responders' status are presented in Figure 3. Out of the 70 patients with complete data, 22 response profiles were identified, with a total of 9 patients responding at each time point for all outcomes, 5 patients who did not respond at all and 20 patients who responded for all outcomes at follow-up regardless of their profile at post-intervention. Details of responders' profile are presented in Figure 4.

### SMS Tracking and Adverse Events

SMS tracking was used to assess side effects for 6 days after the intervention. At day 1, there was a response rate of 84.1% of the 107 patients, with a mean pain of 3.15 (±1.97). At day 6, we observed a response rate of 46.7%, with only 50 patients who kept answering the SMS. Mean pain at day 6 averaged 2.82 (±2.2). Response rates for adverse events averaged 12.1%, with only 13 patients reporting adverse events, such as pain increase (*n* = 10) or muscle aches (*n* = 3).

## Discussion

Based on current evidence suggesting that changes in SMT dosages can lead to changes in physiological and clinical responses, this current study sought out to identify candidate prognostic factors associated with positive short-term treatment responses in chronic thoracic spine pain using SMT. Results indicated that baseline characteristics including expectation of improvement in pain and disability, as well as post-intervention variables such as patients' perceived comfort of SMT and global perceived change were associated with the pain and GPC responder status at post-intervention and/or follow-up. Moreover, the study was designed to identify SMT dosages associated with positive treatment responses. The results showed no association between the biomechanical parameters of SMT and those who responded to treatment for pain, disability levels, as well as the global perceived change score.

### Biomechanical Parameters of SMT

This cohort study explored force-time dosage as a candidate prognostic factor and its potential relationship with clinical outcomes. In a scoping review, Pasquier et al. investigated the current state of scientific knowledge regarding effects of SMT dosages on physiological and clinical responses, and identified studies investigating effects of dosage on clinical outcomes (17). In a recent study, Pagé et al. explored the effect of SMT biomechanical parameters on several outcomes in patients with chronic thoracic pain. Pre-determined doses of peak force, impulse duration, and rates of force applications were used in three different groups and compared to a placebo (18). Their results showed a decrease in pain intensity and disability regardless of dosages in all groups, but no significant dosage effects on clinical outcomes. Overall, these results support the findings of our cohort study and highlight the heterogeneous and relatively scarce nature of current scientific evidence regarding SMT dosages.

Manipulation and mobilization dosage effects have, however, been described for cervical spine treatment. Gudavalli et al. investigated the effect of three different manually delivered cervical traction forces (low, medium and high) on patients experiencing chronic neck pain (39). The results of this pilot study on 48 patients showed that high-force traction significantly improved neck pain, compared to low-force traction, whereas improvements in disability were significantly greater for medium and high-force traction, compared to low-force traction. In a randomized control trial, Snodgrass et al. studied the effect of a manual therapy (SMT) using different doses (90N, 30N, placebo) on both clinical and biomechanical outcomes in patients with neck pain. Their results showed a greater decrease in pain at 4 days in patients treated with 90N spinal mobilization than in those treated with a 30N spinal mobilization, but no difference with patients in the placebo group (40).

### Comfort of SMT

Although our study failed to identify a SMT dosage associated with short-term clinical outcome improvement, this is the first study to investigate how SMT biomechanical parameters and related comfort are associated with short-term clinical outcomes. Results show that patients' perceived comfort of SMT is associated with an improvement in pain immediately following the intervention and with a global perceived change immediately after the intervention and at follow-up. Our results indicate that patients who rated a higher score for comfort of SMT were more likely to have a decrease of pain and to experience a global change of their symptoms during this 1-week post-procedure. Through a Delphi process, O'Donnell et al. investigated what educators and clinicians considered to be important characteristics of the patients and clinicians' positions before and during SMT. Results showed that patient comfort was identified as important, with a high level of agreement (32). In addition to this Delphi, Pasquier et al. investigated associations between objective SMT biomechanical parameters and subjective assessments such as patient comfort perceived by patients, clinicians, and expert assessors (41). Results showed that perceived comfort of the thoracic SMT assessed by the 3 different populations was mostly associated with perceived thrust duration and preload characteristics. The authors also suggest that subjective assessments such as comfort of SMT should be included in manual therapist education and assessment to enhance patients' care.

Comfort is a concept that has been studied in different environments (surgery, geriatrics, or manual care), and for which investigators have developed guidelines and consensus statements to enhance patients' experience (42–44). For instance, to improve perioperative care, it is recommended that patients' experience of comfort be evaluated (42). From a manual care perspective, comfort can also be assimilated to the therapeutic touch. Therapeutic touch has been identified as a modulator of MSD clinical outcomes or even as a placebo, with a positive influence on pain, and is classified as a powerful non-verbal communication tool for therapists (45). Comfort touch has been described in nursing and physiotherapy as a useful strategy to relieve musculoskeletal pain (46–48).

### Expectations of Improvement

Results of the present study show that patients were more likely to have a meaningful decrease in pain intensity when expectation of disability improvement was greater at baseline. In addition, a meaningful GPC at follow-up was associated with greater expectation scores for improvement in pain and disability.

Expectations have been studied recently in the context of SMT. Indeed, Pagé et al. investigated the effect of SMT on outcomes in patients with chronic thoracic pain (18). Exploratory results showed that there was no association between initial expectation and “improved” patients after SMT sessions and initial expectation could not predict patients' response to treatment. Moreover, in a review, expectations have been studied and identified as important in the placebo analgesia process, and seem to be associated with changes in clinical outcomes of a treatment (45, 49). In a study investigating expectations in patients with thoracic spine pain, Bishop et al. investigated patients' expectations of improvement when care is delivered by physical therapists. Their studies investigated the extent to which the patients' expectations, particularly for spinal manipulation, affect clinical outcomes. Their results showed, for patients with low back pain as well as patients with neck pain, that expectations of benefit were greater for manual therapy compared to other therapies. Results also showed that expectations of improvement following SMT was associated with increased short-term recovery and long-term decrease in disability (50, 51).

Cormier et al. explored the association between expectations and clinical outcomes of patients with chronic pain. The authors showed that there is an association between expectations and clinical outcomes following a multidisciplinary treatment approach for chronic pain. Their results showed that pain responder status was associated with greater pretreatment expectations and higher global perception of change after care (27). A systematic review sought to synthesize evidence on the association between expectations and various outcomes in adults with low back pain. The results showed that expectations may be associated with clinically important recovery outcomes, but also that the overall evidence was of low quality (52). Overall, our results indicating that the pain and GPC responder status were linked to more positive expectation of pain improvement or disability prior to an SMT treatment and seem to support the results of previous studies.

### Other Candidate Prognostic Factors

Although patients' characteristics were assessed in this study, none were linked to clinical responses. Anthropometric criteria such as height, weight or BMI do not influence treatment response. Moreover, the influence of anthropometric characteristics on biomechanics parameters has been previously investigated. Mikhail et al. (53) investigated the difference between the force measured at the patient-table interface and the force applied at the clinician-patient interface during thoracic spinal manipulative treatment and mobilization to determine the influence of the manipulation or mobilization characteristics, patients' anthropometry, and muscle activity on this difference. The results showed that the forces measured at the patient-table interface are slightly greater than the forces applied at the clinician-patient interface during thoracic manipulations and mobilizations. In addition, results suggest that anthropometric characteristics and muscle activation do not influence on the difference between the forces at the patient-table and clinician-patient interface (54).

While patients' characteristics were not correlated with any outcome in the model, 20 patients were full responders at the follow-up (patients who met or exceeded MIC for all outcomes variables at follow-up). These 20 patients included 11 women (mean age = 32.9 ± 15.4) and 9 men (mean age = 29.9 ± 9.8) with women presenting significantly higher level of disability at baseline (24.6 ± 16.3 vs. 10.9 ± 5.6; *p* = 0.027). Global responders (*n* = 20) at follow-up had higher expectation for pain improvement and disability, higher GPC score post-intervention and lesser pain at post-intervention compared to non-responders (*n* = 9).

From a clinical standpoint, it is noteworthy to mention that we did not find a strong candidate prognostic factor of treatment response in our study, but defining responders is challenging and could be a reason explaining those difficulties.

### Limitations

One of the main limitations of this observational study is its short-term follow-up. A 7-day period did not allow us to observe changes over a longer period of time in the same patient. It is therefore not possible to establish long-term associations between expectations, SMT comfort and clinical outcomes. Such design does not provide evidence of causation, but certainly informs future studies by identifying SMT candidate prognostic factors.

A second limitation is the loss of patients to follow up and missing data. Despite our efforts to minimize follow-up data loss, a total of 10 patients did not complete any of the follow-up assessment. In addition, the Quebec Back Pain Disability Scale was assessed only at baseline and follow-up because it was deemed irrelevant to evaluate patients' disability changes immediately following spinal manipulative treatment, since it evaluates daily life activities. Functional capacities following SMT could be investigated in future studies through range of motion, strength, stiffness, or other performance-based outcomes (54).

Other limitations include our definition of chronic pain based only on symptom duration and included both persistent and recurrent pain patterns which are sometimes considered as two distinct conditions. Additionally, there is currently no available MIC for thoracic spine pain populations following SMT. However, since MIC is considered both patient and intervention dependent, it was deemed reasonable to use values drawn from low back pain population (35).

Moreover, the study was not designed to test an overall responder model combining pain, disability and global perceived change. Future research should examine these candidate prognostic factors in combination. Finally, interventions were provided by chiropractic students in an educational outpatient clinic under the supervision of expert clinicians. This particular clinical context may limit the overall generalizability of the results.

### Practical and Clinical Applications

Although this study did not highlight any association between SMT force-time profile and treatment responses, it seems to suggest that perceived comfort may be associated with clinical outcomes. Dosage parameters of SMT might therefore be indirectly linked to clinical responses, and patients' preferences in dosage should be considered in future studies. Indeed, a previous study showed that comfort of SMT is associated with thrust duration, preload force as well as drop in preload force (41). Regardless SMT biomechanical parameters, the manual therapist's education and training should emphasize patient preferences and adapt manual techniques in light of previous SMT experiences and perceived comfort of the procedure. Future studies investigating patients' preference including preferred dosages and associated clinical outcomes are warranted. Long-term prognostic factors should also be investigated.

## Conclusion

In conclusion, this exploratory study investigating short-term candidate prognostic factors of positive responses to a spinal manipulation treatment in patients with non-specific thoracic back pain showed that no specific dosage of SMT is associated with short-term clinical responses to treatment. However, expectations of improvement, as well as patient's comfort with SMT and pain change at post-intervention were associated with a positive response to treatment. The study suggests that usual clinical outcomes such as pain and disability may not be the only factors of a positive treatment to consider when establishing prognosis for patient with thoracic spine pain. The result of this study should be considered as a basis for further causation or prediction studies aiming to develop clinical strategies and enhance management of patients with spinal pain.

## Data Availability Statement

The raw data supporting the conclusions of this article will be made available by the authors, without undue reservation.

## Ethics Statement

The studies involving human participants were reviewed and approved by Ethics Committee of the Université du Québec à Trois-Rivières (CER-20-265-10.02) and French Committees of Protection of Persons (19.04.27.61617-2019_45). The patients/participants provided their written informed consent to participate in this study.

## Author Contributions

MP: conception and design of the study's, data collection, data analysis and interpretation, drafting the article, and final approval of the version to be published. JY: data analysis, critical revision of the article, and final approval of the version to be published. AL: conception and design of the study's, data analysis and interpretation, critical revision of the article, and final approval of the version to be published. MD: conception and design of the study's, critical revision of the article, and final approval of the version to be published. All authors contributed to the article and approved the submitted version.

## Funding

MP received a scholarship from the Fondation Chiropratique du Québec for a doctoral thesis.

## Conflict of Interest

The authors declare that the research was conducted in the absence of any commercial or financial relationships that could be construed as a potential conflict of interest. The reviewer PT declared a shared consortium with one of the authors MD at time of review.

## Publisher's Note

All claims expressed in this article are solely those of the authors and do not necessarily represent those of their affiliated organizations, or those of the publisher, the editors and the reviewers. Any product that may be evaluated in this article, or claim that may be made by its manufacturer, is not guaranteed or endorsed by the publisher.
