# Supplementary material for: Factors Associated With Clinical Responses to Spinal Manipulation in Patients With Non-specific Thoracic Back Pain: A Prospective Cohort Study
Source: Front Pain Res (Lausanne). 2022 Jan 6;2:742119. doi: 10.3389/fpain.2021.742119 (PMC8915706; doi:10.3389/fpain.2021.742119)
Supplement: Supplementary file 1 [file Data_Sheet_1.PDF]

Supplementary file 1. Baseline characteristics by pain responder status at post-intervention and follow-up

	Pain responder status *at post-intervention* (n=82)			Pain responder status *at follow-up* (n=74)		
VARIABLES	Responders (n=31)	Non-responders (n=51)	p-value	Responders (n= 49)	Non-responders (n=25)	p-value
**Preload** (N)	186 (140)	163 (110)	0.716⁺	181 (110)	143 (133)	0.151⁺
**Peak force** (N)	485.9 (±184.5)	453.67 (±141.7)	0.376⁻	475.2 (±150.4)	422.4 (±160.7)	0.167⁻
**Thrust duration** (ms)	134 (41)	131 (29)	0.515⁺	128 (26)	134 (31)	0.823⁺
**Rate of force** ($N.s^{-1}$)	2455.9 (±1044.6)	2392.9 (±844.6)	0.765⁻	2471.6 (±978.6)	2307.3 (±891.4)	0.484⁻
**Drop in preload** (N)	25 (48)	23 (41)	0.741⁺	18 (46)	25 (35)	0.381⁺
**Expectation of improvement in pain** (-5 to 5)	3.5 (3)	4 (2)	0.162⁺	4 (2)	3 (2)	0.205⁺
**Expectation of improvement in disability** (-5 to 5)	3.5 (2)	4 (2)	0.452⁺	4 (2)	3 (1)	***0.036**⁺
**Kinesiophobia – Tampa** (/68)	29.6 (±11.4)	29.8(±12.1)	0.939⁻	29.1 (±11.7)	30.5 (±11.6)	0.620⁻
**Level of anxiety** -STAI-YA (/100)	36 (10)	34 (13)	0.524⁺	36 (11)	34 (14)	0.684⁺
**Level of anxiety** - STAI-YB (/100)	39 (9)	37 (17)	0.483⁺	39 (11)	40 (18)	0.592⁺
**Comfort** (0-10)	7.7 (±1.8)	5.8 (±2.2)	***<0.001**⁻	6.79 (±2.15)	6.16 (±2.44)	0.259⁻
**Pain at baseline - NRS** (0-10)	4 (3)	5 (2.4)	0.202⁺	4.3 (2.9)	5 (2)	0.427⁺
**Disability at baseline - QBPS** (/100)	12 (11)	15 (18)	0.259⁺	13.5 (15.5)	15 (18)	0.463⁺
**Pain change at post-intervention**		X		1 (2.5)	0.75 (1.35)	0.085⁺
**GPC at post-intervention** (-5 to 5)		X		2 (1.75)	2 (3)	0.338⁺

** if significant value; ⁺Wilcoxon rank sum test; ⁻T-test*

n = number of patients; SD = Standard Deviation; STAI= State-Trait-Anxiety Inventory; NRS= Numeric Rating Scale; QBPS= Quebec Back Pain Scale *GPC= Global perceived change; IQR = Interquartile Range*

Mean (±SD) are presented for normally distributed data and Median (IQR) are presented for non-normally distributed data
