# Supplementary material for: Factors Associated With Clinical Responses to Spinal Manipulation in Patients With Non-specific Thoracic Back Pain: A Prospective Cohort Study
Source: Front Pain Res (Lausanne). 2022 Jan 6;2:742119. doi: 10.3389/fpain.2021.742119 (PMC8915706; doi:10.3389/fpain.2021.742119)
Supplement: Supplementary file 2 [file Data_Sheet_2.PDF]

Supplementary file 2. Baseline characteristics by disability responder status at follow-up

	Disability responder status ***at follow-up (n=94)***		
VARIABLES	**Responders (n=41)**	**Non-responders (n=53)**	**p-value**
**Preload** (N)	183 (117)	152 (101)	0.287^{+}
**Peak force** (N)	468.2 (±171.0)	432.7 (±137.0)	0.267^{-}
**Thrust duration** (ms)	133 (32)	124 (25.0)	0.099^{+}
**Rate of force** (N.s-^{1})	2330.3 (±891.9)	2370.6 (±857.9)	0.824^{-}
**Drop in preload** (N)	21 (42)	25 (45)	0.489^{+}
**Expectation of improvement in pain** (-5 to 5)	4 (2)	4 (2)	0.394^{+}
**Expectation of improvement in disability** (-5 to 5)	4 (1.5)	3 (2.2)	0.324^{+}
**Kinesiophobia – Tampa** (/68)	28.8 (±11.1)	29.69 (±11.1)	0.701^{-}
**Level of anxiety** -STAI-YA (/100)	33.0 (13)	34 (14)	0.516^{+}
**Level of anxiety** - STAI-YB (/100)	38 (10)	43 (16)	0.473^{+}
**Comfort** (0-10)	6.60 (±2.47)	6.45 (±2.44)	0.768^{-}
**Pain at baseline - NRS** (0-10)	4 (2.75)	5 (2)	0.080^{+}
**Disability at baseline - QBPS** (/100)	15 (17)	12 (11)	0.281^{+}
**Pain change at post-intervention**	1 (1.25)	1 (2)	0.807^{+}
**GPC at post-intervention** (-5 to 5)	2 (2.7)	2 (3)	0.832^{+}

$^{+}$*Wilcoxon rank sum test;* $^{-}$*T-test*

n = number of patients; SD = Standard Deviation; STAI= State-Trait-Anxiety Inventory; NRS= Numeric Rating Scale; QBPS= Quebec Back Pain Scale; GPC= Global perceived change; IQR = Interquartile Range

Mean (±SD) are presented for normally distributed data and Median (IQR) are presented for non-normally distributed data
