# Supplementary material for: Factors Associated With Clinical Responses to Spinal Manipulation in Patients With Non-specific Thoracic Back Pain: A Prospective Cohort Study
Source: Front Pain Res (Lausanne). 2022 Jan 6;2:742119. doi: 10.3389/fpain.2021.742119 (PMC8915706; doi:10.3389/fpain.2021.742119)
Supplement: Supplementary file 3 [file Data_Sheet_3.PDF]

Supplementary file 3. Baseline characteristics by GPC responder status at post-intervention and follow-up

	GPC responder status **at post-intervention (n=105)**			GPC responder status **at follow-up (n=94)**		
VARIABLES	**Responders (n=68)**	**Non-responders (n=37)**	**p-value**	**Responders (n=68)**	**Non-responders (n=26)**	**p-value**
**Preload** (N)	162.5 (120)	161 (101)	0.707[+]	161.5 (125)	155.5 (89)	0.796[+]
**Peak force** (N)	457.8 (±166.5)	439.3 (±139.0)	0.566[-]	450.7 (±153.0)	435.5 (±154.4)	0.667[-]
**Thrust duration** (ms)	127.5 (27)	128 (32)	0.869[+]	126 (25)	128.0 (41)	0.706[+]
**Rate of force** ($N.s^{-1}$)	2362.1 (±883.7)	2355.9 (±860.4)	0.972[-]	2434.6 (±926.1)	2219.3 (±799.8)	0.298[-]
**Drop in preload** (N)	18 (41)	30 (44)	0.077[+]	18 (47.5)	24.5 (41)	0.313[+]
**Expectation of improvement in pain** (-5 to 5)	4 (2)	4 (1.5)	0.325[+]	4 (2)	3 (2)	***0.005**[+]
**Expectation of improvement in disability** (-5 to 5)	4 (2)	3 (1.5)	***0.022**[+]	4 (2)	3 (2)	***0.017**[+]
**Kinesiophobia – Tampa** (/68)	29.4 (±11.4)	29.3 (±10.7)	0.976[-]	28.9 (±11.5)	30.2 (±9.9)	0.622[-]
**Level of anxiety** -STAI-YA (/100)	36 (12)	33 (13)	0.575[+]	36 (14)	33 (14)	0.626[+]
**Level of anxiety** - STAI-YB (/100)	37.5 (14)	40 (15)	0.537[+]	38 (11)	43 (19)	0.517[+]
**Comfort** (0-10)	7.09 (±2.3)	5.5 (±2.3)	***<0.001**[-]	6.9 (±2.2)	5.4 (±2.5)	***0.006**[-]
**Pain at baseline - NRS** (0-10)	4.76 (2.65)	4.65 (3)	0.550[+]	4.2 (2.76)	5 (2)	0.327[+]
**Disability at baseline - QBPS** (/100)	13.5 (14)	9 (13.5)	0.348[+]	13.5 (13.5)	18 (8)	0.211[+]
**Pain change at post-intervention**		X		1 (2.7)	0.85 (1.65)	0.272[+]
**GPC at post-intervention** (-5 to 5)		X		3 (2.5)	1 (2)	***<0.001**[+]

** if significant values; [+]Wilcoxon rank sum test; [-]T-test*

n = number of patients; SD = Standard Deviation; STAI= State-Trait-Anxiety Inventory; NRS= Numeric Rating Scale; QBPS= Quebec Back Pain Scale; GPC= Global perceived change; IQR = Interquartile Range

Mean (±SD) are presented for normally distributed data and Median (IQR) are presented for non-normally distributed data
